# Virtual consultation for actinic keratosis

**DOI:** 10.3399/bjgpopen20X101126

**Published:** 2020-09-23

**Authors:** Sukhjit Dhariwal, Tushar Hari, Kamal Kaur, Chamandeep Thind, Alison Bedlow, Bruce C Gee, Simon Tso

**Affiliations:** 1 Specialist Registrar Dermatology, Jephson Dermatology Centre, South Warwickshire NHS Foundation Trust, Warwick, UK; 2 Medical Student, The University of Buckingham Medical School, Buckingham, UK; 3 GP, Horseley Health Surgery and Tandon Medical Centre, Tipton, UK; 4 Consultant Dermatologist, Jephson Dermatology Centre, South Warwickshire NHS Foundation Trust, Warwick, UK; 5 Consultant Dermatologist, Jephson Dermatology Centre, South Warwickshire NHS Foundation Trust, Warwick, UK; 6 Consultant Dermatologist and Lead Skin Cancer Clinician, Jephson Dermatology Centre, South Warwickshire NHS Foundation Trust, Warwick, United Kingdom

**Keywords:** Dermatology, Hospital referrals, Keratosis, Actinic, Primary health care, Secondary care, General practitioners, General practice, Remote consultation

## Introduction

Actinic keratoses (AK) are keratotic lesions presenting on chronically ultraviolet-exposed skin. The rate of malignant progression of a single AK to squamous cell carcinoma (SCC) remains uncertain, with reports ranging from 0.025%–20%.^1,2^ Patients receiving long-term immunosuppressive medications are at higher risk of developing AK.^3^ The estimated prevalence of AK is 19%–24% of individuals aged >60 years in the UK.^3^ Research suggests between 25%–70% of AK may spontaneously regress in a 1–4 year period.^3^ A Dutch qualitative study highlighted that some primary care clinicians’ principal approach to managing AK was treatment with cryosurgery, or referral to secondary care with patient-driven follow-up care.^4^ This article aims to inform GPs on the management of AK based on the British Association of Dermatologists (BAD) guidelines, the authors’ opinions on effective virtual consultations on AK, and when to refer patients to secondary care.

## Clinical presentation of actinic keratosis

AK are often asymptomatic but can be sore or itchy. The presentation of AK is classified into three grades according to the BAD.^3^ Grade 1 (mild) AK are minimally scaly patches, grade 2 (moderate) are moderately scaly patches, and grade 3 (severe) are hyperkeratotic lesions.^3^ Confluent areas of AK (field changes) signifies extensive actinic damage.^3^ International guidelines have classified four different patient groups for treatment: patients with single AK lesions (1–5 AK per field or body region), patients with multiple AK lesions (≥6 AK lesions on one body region or field), patients with field cancerisation (≥6 AK lesions in one body region or field), and patients with concomitant immunosuppression.^5^

## Management approach to actinic keratoses

The BAD guideline indicates that treatment for AK *‘* *is not universally required on the basis of preventing progression into squamous cell carcinoma* *’*.^3^ In our practice, some patients with asymptomatic grade 1 and 2 AK had made an informed decision to self-monitor the lesions. In this situation, we recommended the use of keratolytic emollients, and provided an AK patient information leaflet, sun protection advice, and safety net advice on when they should seek medical attention. Regular use of sunscreen can reduce the development of AK.^6^

The indications, treatment regimes, possible complications, and success rates of prescription only-topical therapies and other treatments available are presented in Tables 1 and 2, respectively. Meta-analysis of relative efficacy of treatments for AK of the face or scalp showed photodynamic therapy (PDT) is more likely to achieve total clearance from AK followed by 5-flurouracil 0.5% cream, imiquimod 5% cream for 4 weeks, cryotherapy, and diclofenac 3% gel (5-flurouracil 5% cream was excluded from the meta-analysis).^7^ The choice of treatment depends on patient preference, the burden of the treatment, body site, patient comorbidities, side effects, whether field treatment is required, maximum size of treatment field per treatment cycle, treatment costs, duration of treatment, and the clinician’s experience. We recommend clinicians familiarise themselves with their area prescribing committee’s policy in relation to the costs of treatment, treatment regimes, treatment availability, and treatment pathways, as this may vary in different settings in different countries.

**Table 1.** Prescription only-topical therapies for actinic keratosis^a3^

	**Regime**	**Indication**	**Possible complications/ Tips on treatment**	**Success rate**
**Diclofenac 3% gel**	Twice daily for 60–90 days, maximum 8 g per day.	Grade 1 AK with or without field change.	Pruritus (41%), rash (40%), xerosis, and erythema.**Caution** – Usually well tolerated but after the course has been completed AK tend to recur.	19%–70%
**5-flurouracil 5% cream**	Once daily for 3–4 weeks or twice daily for up to 3 weeks (lower limbs may require longer treatment duration).Cycle can be repeated a month later if AK persists.	All grades of AK with or without field change.	Local irritation, pain, dryness, erythema, erosion, or oedema.**Tip** - Test on a small area first and then, if tolerated, treat further sections of field damage.**Tip** - Side effects can be minimalised by reducing the frequency of application, taking short breaks in the course of treatment or, if needed, applying topical corticosteroid such as clobetasone butyrate.**Tip** – Application of urea-based emollients before applying 5-flurouracil can enhance the effect.**Caution** – Ulceration and slow healing are possible on lower legs.	70%–78%
**5-flurouracil 0.5% cream with salicylic acid 10%**	Once daily for up to 12 weeks (maximum area of treatment is 25 cm^2^ at one time).	Grade 2 and 3 AK without field change.Moderately thick hyper-keratotic AK lesions.	Local inflammatory reaction.**Caution** - Patients with severe hand arthritis or limited hand dexterity will find it difficult to use the applicator to apply the treatment and peel off the film.	55%–77%
**Imiquimod (5% cream**)	Apply at night and washed off 8 hours later, three times a week for 4–6 weeks.	Non-hyper-keratotic and non-hypertrophic AK on face and scalp in immunocompetent patients.Second line treatment for AK resistant to 5-flurouracil.	Severe erythema, scabbing, crusting, erosions, ulceration, and flu-like symptoms (rare).**Tip** - Side effects can be minimalised by reducing the frequency of application, taking short breaks in the course of treatment, using lower strength 3.75% imiquimod cream, or applying a topical corticosteroid such as clobetasone butyrate.	50%–84%

a All topical treatments, except for imiquimod 5% cream, may be initiated by GPs depending on the local area prescribing policy. Imiquimod 5% cream is a specialist-initiated treatment in the UK. All treatments can be applied by patients at home by themselves or with help from family. Please refer to manufacturer’s instructions as to the maximum area of application of the treatment.

**Table 2.** Other treatments for actinic keratosis^3^

	**Regime**	**Indication**	**Complications/ Tips on treatment**	**Success rate**
**Cryosurgery (liquid nitrogen) as a lesion-directed treatment^a^**	Single cycle	Effective on all sites and all grades of AK without field change. Particularly effective for hyperkeratotic AK.	Soreness, blistering, hair loss, scarring, infection, and pigmentary skin changes.**Caution** - Slow healing and ulceration are possible below the knee, on the scalp, or on the pinna especially in older patients.**Caution** – Can be difficult to use near mouth and eyes.	39%–88%
**Photodynamic therapy (PDT) as a lesion-directed or field treatment**	Photosensitising topical cream/gel applied under occlusion followed by illumination of the treatment field with a laser or non-laser light (eg, blue light or red light; conventional PDT or natural sunlight; daylight PDT).	Conventional PDT – suitable for Grade 2 and 3 AK with or without field change.Daylight PDT – suitable for extensive grade 1 AK with field change.Only available in secondary care.	Pain, initial erythema, burning sensation and crusting followed by pigmentary change and ulceration.**Tip** - Suitable for scalp and below knee lesions with a lesser risk of causing poor wound healing as compared to cryosurgery and 5-Flurouracil.	69%–93%
**Skin surgery^b^ as a lesion directed treatment**	Curette and cautery (2 cycles) or excision biopsy.	If diagnosis uncertain, histology is required, large keratin horns, grade 3 AK and AK resistant to therapies listed in Table 1.	Infection, bleeding, scarring, poor wound healing and pain.**Caution** - Slow healing and ulceration are possible below the knee especially in elderly patients.	No clinical trials

Photodynamic therapy is performed in secondary care. Skin surgery and cryosurgery can be performed by GPs with prior training. ^a^5 seconds of total treatment time achieves a 39% cure rate and over 20 seconds of total treatment time achieves 83% cure rate. ^b^Electrodessication and curettage provides 95–99% clearance for lesion-directed therapy for hyperkeratotoic AK as other therapies cannot penetrate as deep as electrodessication and curettage.

The treatments listed in Table 1 are expected to cause varying degrees of skin inflammation (typically diclofenac 3% gel causes less skin inflammation than 5-fluorouracil, which causes less than imiquimod) and the residual inflammatory response could take up to 4 weeks (or longer, especially on lower legs) after stopping treatment to fully resolve. Exaggerated response from diclofenac 3% gel could be due to allergic contact dermatitis. If patients could not tolerate the extent of expected inflammatory response from these prescription only-topical therapies, then the treatment should be witheld and the use of topical corticosteroids such as clobetasone butyrate and emollients may be used to hasten the recovery. Patients with genuine concerns about topical therapies causing significant inflammation near delicate body sites (eyes, mouth, and areas of skin erosion), who declined other management options, may be advised to use the therapies more cautiously with a reduced frequency regime (for example, half the frequency or half the duration of treatment) on an off-license basis. In our experience, this is less effective in clearing AK as compared to the licensed regimes and readers should note that this is expert advice only, with no clinical trial evidence to support this practice. Patients should be given an information leaflet with images of possible post-treatment inflammatory responses in order to address patient expectations and anxiety related to inflammation. Topical 5-fluorouracil is toxic to pets and patients should be warned not to let their pets ingest the treatment through licking the patient’s skin.^8^

## Virtual consultations

Virtual consultations have benefits but also risks^9^ as clinicians cannot palpate the skin to assess for potential invasion, risking misdiagnosis of a skin cancer. In primary care it may be appropriate to offer an initial face-to-face assessment for accurate risk assessment, diagnosis, and discussion of treatment options with patients presenting with AK. Virtual consultation could then be employed for subsequent clinician-led or patient-led follow-up to discuss treatment outcome and management of expected side effects from treatments.

Virtual consultations should ideally be supplemented with good quality photographs — taken by patients, carers, or professionals under good lighting, clearly showing the anatomical location (far view) and close up views of the skin lesion site next to a ruler — which should be sent to a clinician in advance of a virtual consultation. Photographs taken at regular time intervals could be useful to demonstrate the evolution of the lesion before, during, and after treatments. Clinicians must not rely on patients to palpate around the lesion to tell a clinician whether the underlying skin is indurated. AK is an epidermal lesion, thus full resolution of an AK means the patient should report the treatment site returning to normal skin texture without residual scaling. However, some degree of pigmentary skin change and scarring following treatments may occur, especially following cryosurgery treatment. Topical treatments (Table 1) can all be used by patients at home with help from family members wearing protective gloves to apply treatments if needed. Patients should be educated on AK by explaining what they should expect during and after the treatment, and what warning signs to look out for. They should be provided with patient information leaflets and be aware of the importance of wearing sun protection. Clinicians should always organise for the patient to be assessed face-to-face if they are in any doubt about the diagnosis or whether AK have fully cleared.

## Referral to secondary care

**Figure 1.**
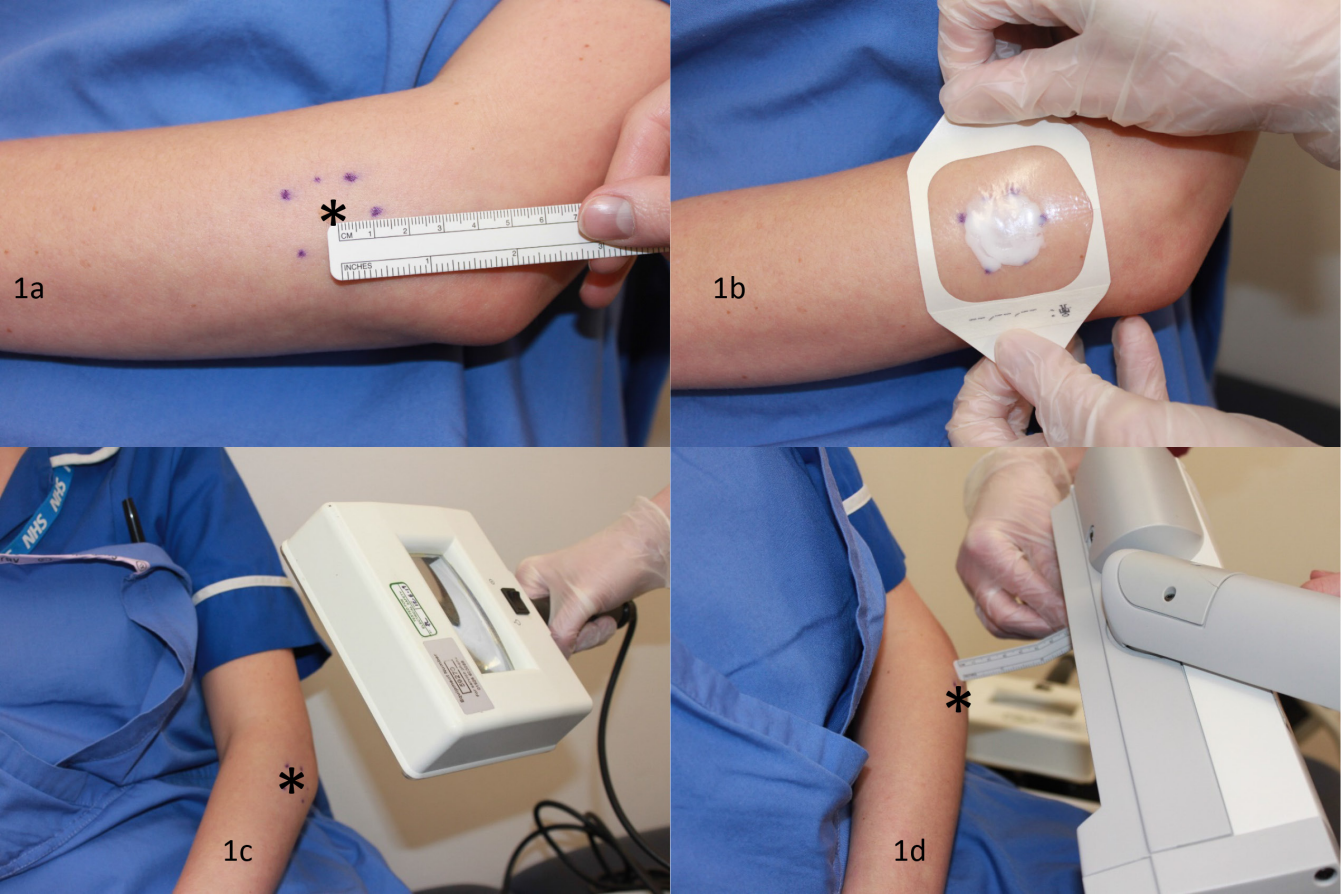
Conventional photodynamic therapy. Image shows how conventional photodynamic therapy is performed on a simulated patient. *denotes the lesion site. 1a) The lesion site is marked with a 1cm peripheral margin followed by descaling of the treatment zone with a blade and 1b) the application of a photosensitising agent built up to 1mm thickness which is left on for a minimum of 3 hours under occlusion with a dressing. 1c) The treatment zone should fluoresce pink when examined under a Woods lamp. 1d) A conventional PDT machine is stationed between 5–8cm away from the treatment zone site shining a light (at 37 joules/cm²) at the treatment zone for approximately 7 minutes.

We encourage referral to secondary care if there is diagnostic uncertainty, treatment resistant AK, or patients with grade 3 AK to exclude a SCC. Organ transplant patients on long term immunosuppressive treatments with lesions due to actinic damage can be referred to secondary care for long-term skin cancer surveillance. Patients with widespread actinic damage could benefit from risk assessment, patient education, and field treatment in secondary care. We wish to highlight that PDT (Figure 1) is a treatment performed in secondary care capable of treating a field size of 16 × 6 cm (conventional PDT) or the whole scalp (daylight PDT). Multiple conventional PDT machines can be used at the same treatment session to treat multiple body sites.

## Conclusion

We are of the opinion that patients with grade 1 and 2 AK can be managed effectively in primary care, and patients with AK that is challenging to manage can be referred onwards for secondary care assessment.
